# Nanomaterials for Sensory Systems—A Review

**DOI:** 10.3390/bios15110754

**Published:** 2025-11-11

**Authors:** Andrei Ivanov, Daniela Laura Buruiana, Constantin Trus, Viorica Ghisman, Iulian Vasile Antoniac

**Affiliations:** 1 Interdisciplinary Research Centre in the Field of Eco-Nano Technology and Advance Materials CC-ITI, Faculty of Engineering, “Dunarea de Jos” University of Galati, 47 Domneasca, 800008 Galati, Romania; andrei.ivanov@ugal.ro (A.I.); constantin.trus@ugal.ro (C.T.); viorica.ghisman@ugal.ro (V.G.); 2 Faculty Materials Science & Engineering, National University Science & Technology Politehnica Bucharest, 313 Splaiul Independentei, Dist 6, 060042 Bucharest, Romania; antoniac.iulian@gmail.com

**Keywords:** nanomaterials, biosensors, food quality, food safety, smart packaging

## Abstract

Nanotechnology offers powerful new tools to enhance food quality monitoring and safety assurance. In the food industry, nanoscale materials (e.g., metal, metal oxide, carbon, and polymeric nanomaterials) are being integrated into sensory systems to detect spoilage, contamination, and intentional food tampering with unprecedented sensitivity. Nanosensors can rapidly identify foodborne pathogens, toxins, and chemical changes that signal spoilage, overcoming the limitations of conventional assays that are often slow, costly, or require expert operation. These advances translate into improved food safety and extended shelf-life by allowing early intervention (for example, via antimicrobial nano-coatings) to prevent spoilage. This review provides a comprehensive overview of the types of nanomaterials used in food sensory applications and their mechanisms of action. We examine current applications in detecting food spoilage indicators and adulterants, as well as recent innovations in smart packaging and continuous freshness monitoring. The advantages of nanomaterials—including heightened analytical sensitivity, specificity, and the ability to combine sensing with active preservative functions—are highlighted alongside important toxicological and regulatory considerations. Overall, nanomaterials are driving the development of smarter food packaging and sensor systems that promise safer foods, reduced waste, and empowered consumers. However, realizing this potential will require addressing safety concerns and establishing clear regulations to ensure responsible deployment of nano-enabled food sensing technologies. Representative figures of merit include Au/AgNP melamine tests with LOD 0.04–0.07 mg L^−1^ and minute-scale readout, a smartphone Au@carbon-QD assay with LOD 3.6 nM, Fe_3_O_4_/DPV detection of Sudan I at 0.001 µM (linear 0.01–20 µM), and a reusable Au–Fe_3_O_4_ piezo-electrochemical immunosensor for aflatoxin B1 with LOD 0.07 ng mL^−1^ (≈15 × reuse), alongside freshness labels that track TVB-N/amine in near-real time and e-nose arrays distinguishing spoilage stages.

## 1. Introduction

Ensuring food safety and quality through effective monitoring is a critical challenge for the global food industry. Traditional analytical methods for detecting spoilage or contaminants in foods (such as microbiological culturing or chemical assays) are often laborious, time-consuming, and require specialized equipment or expertise, which limits their practicality for real-time quality control [1]. In recent years, nanotechnology—the science of manipulating materials at the nanometer scale—has emerged as a promising solution to these limitations. By virtue of their tiny size and large surface area, nanomaterials can exhibit enhanced reactivity and novel optical or electrical properties that enable them to act as extremely sensitive probes of chemical and biological phenomena in foods [2]. The incorporation of such nanomaterials into sensory systems (sensors and detection devices) is revolutionizing how we monitor food quality from farm to fork.

Nanotechnology is being applied across a wide range of food industry domains, including packaging, processing, and safety testing [3]. Nanomaterial-based sensors (nanosensors) can detect trace levels of foodborne pathogens, toxins, allergens, or chemical adulterants that might be present in food products [4]. For example, nanosensors have been developed to quickly detect microbial contamination (e.g., pathogenic E. coli or Salmonella on foods) and hazardous chemicals or pesticides, offering a level of speed and sensitivity beyond conventional diagnostics [5,6]. In parallel, nanoengineered packaging materials are being designed to actively maintain and indicate the condition of the food: these “smart” packaging systems can respond to changes in the food’s environment (such as the accumulation of spoilage gases or changes in pH) and signal the freshness of the product to consumers or quality control personnel [7]. Some nanocomposite packaging also includes active functions—for instance, embedding antimicrobial nanoparticles in packaging films to inhibit the growth of spoilage microbes and extend shelf-life [8]. Nanotechnology-driven approaches offer dual benefits: improving food quality detection and enhancing packaging protection [9,10].

Scope and definitions. We cover both biosensors (with bio-recognition elements: enzymes, antibodies, aptamers) and physicochemical nanosensors (plasmonic, chemiresistive, catalytic, and photo/electrochemical) that operate without biological receptors. Unless specified, “sensor” refers to either category; when bio-recognition is central, we use “biosensor”.

Recent trends point to smartphone-integrated assays, RFID/NFC-printed nanosensor tags, and active/indicator films moving from the laboratory to pilot and first deployment, particularly in perishables tracking and on-site adulterant detection. Some examples include antimicrobial nano-packaging in the meat/fruit and vegetable supply chains, freshness indicator stickers/labels visible to the end-user, and smartphone-scannable hydrogel/colorimetric sensors for measuring TVB-N or individual amines in seconds–minutes. These research-to-market drivers are supported by printed-electronics proof-of-concepts and e-nose prototypes for shelf-life control.

Early research and development in this field has yielded a variety of nanomaterials applicable to food sensory tasks. These include metal nanoparticles (such as gold and silver) for colorimetric or optical biosensors, magnetic nanoparticles for capturing and separating contaminants, carbon-based nanostructures (like carbon nanotubes and graphene) for electrical and electrochemical sensing, and ceramic or metal oxide nanoparticles (e.g., ZnO, TiO_2_) for gas sensing and antimicrobial action [11]. More complex nanostructures like quantum dots and dendrimers have also been explored for sensitive fluorescence-based detections [11,12]. Additionally, the food industry has begun to utilize nanotechnology in the form of nano-encapsulation systems (nanoemulsions, nanoliposomes, etc.), which, while not sensors per se, can improve the delivery of bioactive ingredients and indicators in foods and packaging [11,13]. These developments highlight the interdisciplinary nature of nanofood research, bridging materials science, chemistry, biology, and food technology.

This review provides a detailed examination of “Nanomaterials for Sensory Systems in the Food Industry.” In the sections that follow, we first classify the types of nanomaterials used in food sensory applications and describe their key properties and roles. We then discuss specific applications: how nanosensors are employed to detect food spoilage and intentional food tampering, with examples ranging from pathogen detection to the sensing of spoilage gases and chemical adulterants, and how smart packaging leverages nanotech for real-time condition monitoring. The advantages conferred by nanomaterials—such as heightened sensitivity, faster response, and multifunctionality—are highlighted, along with the current toxicological and regulatory considerations that accompany the use of such novel materials in contact with foods. Finally, we outline future perspectives and research directions needed to address challenges and fully realize the potential of nanomaterials in food industry sensory systems. By consolidating findings from the latest literature, including both the provided sources and other recent studies, this review aims to inform scientists, industry stakeholders, and regulators about the state-of-the-art and the responsible pathway forward for nanotechnology in food sensory applications.

To ensure comprehensive coverage, we integrated >70 peer-reviewed sources (2010–2025) emphasizing recent smartphone-readable, electrochemical, and chemiresistive platforms, and collated quantitative exemplars to enable direct cross-study comparison.

**Figure 1:**
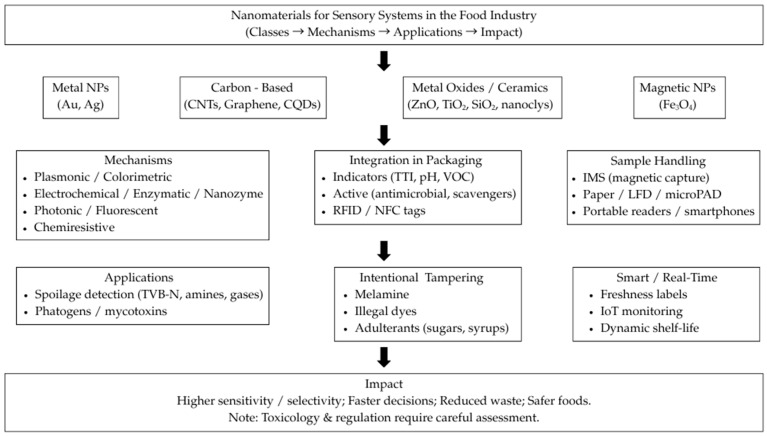
TOC.

A graphical overview of the review’s structure is provided in Figure 1, linking nanomaterial classes to sensing mechanisms and food applications, and highlighting benefits alongside safety/regulatory considerations.

Search strategy and selection criteria. We searched Scopus/Web of Science/MDPI (2010–2025) for nano-enabled food sensing (spoilage markers, adulterants, intelligent packaging, real-time monitoring). We prioritized studies reporting LOD/linear range/response time/selectivity, portable/readout-ready formats (smartphone, μPAD, SPCE), and representative mechanisms (plasmonic, electrochemical, chemiresistive/photonic). The reviews helped contextualize trends; non-food matrices, duplicates, and studies lacking primary performance data were deprioritized.

## 2. Types of Nanomaterials Used in Sensory Systems

Nanomaterials encompass a broad class of substances that have at least one dimension in the nanometer range (approximately 1–100 nm). At this scale, materials often exhibit unique optical, electrical, mechanical, or chemical properties that differ from their bulk counterparts. In the context of food sensory systems, a variety of nanomaterials have been investigated and applied, each offering distinct advantages for sensing or preserving food quality. Table 1 provides an overview of major nanomaterial categories and examples of their use in food industry sensory applications. Below, we discuss each category in detail.

Each type of nanomaterial in Table 1 plays a role in building more responsive and informative food sensing systems. It should be noted that these categories are not mutually exclusive—many practical sensor designs combine multiple nanomaterials. For example, a nanosensor could use a hybrid material like a magnetic particle coated with gold (combining magnetic separation with plasmonic detection), or a graphene sheet decorated with silver nanoparticles to synergize electrical and antimicrobial functions. The design of nanomaterials is often tuned for the specific food matrix and analyte of interest. Organic nanomaterials (like lipid or polymer nanoparticles) may be preferred in edible or direct-contact applications due to potentially lower toxicity, whereas inorganic nanomaterials (metal, oxide, and carbon) are widely used in external sensing devices or packaging inserts for their robustness and strong signal outputs [11,25].

Author Perspective: In reality, the selection of material must be decided by the food matrix (e.g., aqueous juices vs. high-fat dairy), analyte of interest (gases/biogenic amines, small organic adulterants, pathogens, or toxins), and required readout (eye-visual vs. instrumented electrochemical/photonic). For in-package or direct food-contact applications, oxide/ceramic fillers and functional polymers are typically selected on the basis of established barrier and safety profiles, whereas carbon-based platforms (carbon dots, graphene/CNT electrodes) are optimally suited for portable electrochemical or fluorescence sensing. Magnetic nanoparticles are optimally used upstream for capture and clean-up of the sample prior to detection. Green synthesis pathways and surface passivation should be used wherever possible to minimize safety concerns and to allow regulatory approval. This practical environment allows mapping of each nanomaterial class to value-maximizing application while minimizing integration risks.

**Figure 2:**
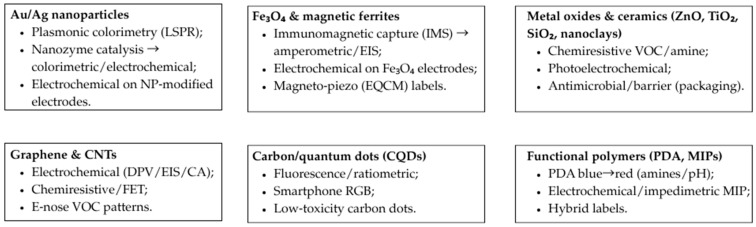
Representative nanomaterial classes and sensing roles in food analysis.

Building on Table 2, Figure 2 maps six representative nanomaterial classes to their principal sensing roles in food analysis: (a) Au/Ag nanoparticles—plasmonic colorimetry (LSPR shift/aggregation) and nanozyme-assisted colorimetric/electrochemical readouts; (b) Fe_3_O_4_/ferrites—immunomagnetic capture (IMS) boosting amperometric/impedimetric signals and magneto-piezo (EQCM) labels; (c) metal oxides and ceramics—chemiresistive VOC/amine sensing and photoelectrochemical enhancement with antimicrobial/barrier functions; (d) graphene/CNTs—electrochemical and chemiresistive/FET platforms (DPV/EIS/chronoamperometry) for freshness markers and adulterants; (e) carbon/quantum dots—fluorescent/ratiometric probes with smartphone-readable RGB quantification; and (f) functional polymers (PDA, MIPs)—PDA blue→red transitions and impedimetric/electrochemical MIP channels.

## 3. Methods of Obtaining Nanoparticles/Nanomaterials

Nanoparticle and nanomaterial synthesis is a fundamental component of applied nanotechnology research and practice, particularly in food-based sensory platforms. The capacity for syntheses of nanostructures with precisely defined size, morphology, and functionality is vital, as these have a direct bearing upon their reactivity, stability, and biocompatibility [26,27]. The approaches are usually categorized as a set of bottom-down and bottom-up methods, supplemented by biological and hybrid methods, designed to marry exactness with feasibility [26,28].

### 3.1. Top-Down Approaches

Top-down approaches are based on breaking down bulk material by physical or chemical means into particles in the nanoscale. These methods are relatively mature, although frequently often limited by irregularities of particle shape and heavy energy requirements [29,30].

- Mechanical milling is the most popular technique in which bulk powders are reduced to nanoparticles by grinding. Though easy and inexpensive, usually it produces wide size distributions and adds defects to the structure [26,28].
- Lithography: Frequently applied in electronics, lithographic techniques utilize optical, electron-beam, or nanoimprint means to outline nanoscale patterns. Despite their accuracy, their expense and complexity place constraints upon their applications in food-based uses [28].
- Laser ablation: Consists of irradiation of a solid target with high-power laser pulses in a liquid. The fast vaporization and condensation provide nanoparticles free of chemical impurities, ready for biomedical and food-associated sensing [27,29].

Advantages: Precise control in some methods; scalability.

Limitations: Energy-intensive; expensive; and poorly suited for broad-spectrum food applications [29,30].

### 3.2. Bottom-Up Approaches

Bottom-up approaches assemble nanomaterials molecule by molecule or atom by atom. These have a greater capability to tune size, morphology, as well as crystallinity, so a larger variety of applications is possible in food applications [28,30].

- Chemical reduction: Metallic nanoparticles such as silver, gold, and copper are synthesized by a reduction of their respective salts through a chemical agent (such as sodium borohydride). These metallic particles have broad potential use in antimicrobial packaging [31,32].
- Sol–gel process: This process involves the hydrolysis and condensation of metal alkoxides or salts, and produces oxides, for instance, SiO_2_ or TiO_2_, of controlled porosity and surface chemistry [28,33].
- Hydrothermal/solvothermal synthesis: This process is carried out in closed reactors at higher temperatures and pressures, where there is accurate control over the size distribution and morphology of nanoparticles [28,31].
- Green synthesis employs natural reducing and stabilizing agents such as plant extracts, polysaccharides, or microbial metabolites. This green approach to synthesis complies with food industry standards by avoiding toxic reagent use [30,32,34].

Advantages: Enhanced structure control, scalability, and reduced energy consumption [28,34].

Limitations: Some methods still require dangerous precursors or solvents [31,34].

### 3.3. Biological Methods

Biological or “green” methods of synthesis utilize the natural biochemical reactions occurring in living organisms.

- Microbial synthesis: bacteria and fungi have been able to decrease metallic ions to nanoparticles through enzymatic reduction and produce stable particles with biofunctional surfaces [35,36].
- Plant-mediated synthesis: Polyphenols, alkaloids, and terpenoids in plant extracts serve as reducing as well as capping agents naturally. The method is inexpensive, eco-friendly, as well as results in food-grade compatible nanoparticles [35,36].
- Enzyme-mediated synthesis: specific enzymes, such as nitrate reductase, facilitate the reduction of metallic ions to nanostructures in mild environmental conditions [36,37].

These methods are perceived as being eco-friendly and specifically appropriate for food packing, biosensors, and preservation systems [36,37].

### 3.4. Deposition Techniques

Thin films or layers that have nanoscale properties are often produced by deposition methods for applications associated with sensor integration or packaging.

- Physical Vapor Deposition (PVD): This method involves vaporizing material in a vacuum and condensing it onto a previously prepared substrate. Produces thin, homogenous films of metals or oxides [38].
- Chemical Vapor Deposition (CVD): This method is based upon chemical reactions from gaseous precursors in forming solid films onto a substrate. CVD is applied to create carbon nanotubes, graphene, or metal oxides used in sensor platforms [38].

These methods are perceived as being eco-friendly and specifically appropriate for food packing, biosensors, and preservation systems [38,39], see Table 3.

## 4. Applications of Sensory Systems in Food Industry

Nano-enabled food sensors typically transduce via (i) optical/plasmonic (colorimetry, fluorescence/ratiometric), (ii) electrochemical (amperometry, DPV, EIS, EQCM), and (iii) chemiresistive/pattern-recognition (gas arrays/e-noses) modalities. Table 2 and Figure 2 map nanomaterial classes to these channels; S4 then applies this framework to freshness/spoilage and adulteration with quantitative comparisons.

Polydiacetylene (PDA) has emerged as a versatile platform for the development of sensory systems in the food industry due to its unique optical transduction, straight-forward functionalization, and stability under variable conditions. PDA-based sensors have been widely explored in two major directions:

Food quality monitoring: PDA vesicles and films functionalized with specific receptors enable detection of freshness-related compounds. Volatile amines such as ammonia, histamine, and propylamine are widely monitored in fish and meat products [40,41]. Ethylene is also a critical indicator for ripening processes in fruits and vegetables, and PDA systems provide simple, colorimetric strategies for its detection [41].

Food contaminant detection: PDA systems can be tailored to identify harmful components and residues. Reported applications include the rapid detection of bacterial pathogens, screening for antibiotic residues in dairy and meat, identification of pesticide traces (e.g., cypermethrin, chlorpyrifos) using PDA + AgNP composites [26], and the monitoring of heavy metals and other toxic compounds [42].

**Figure 3:**
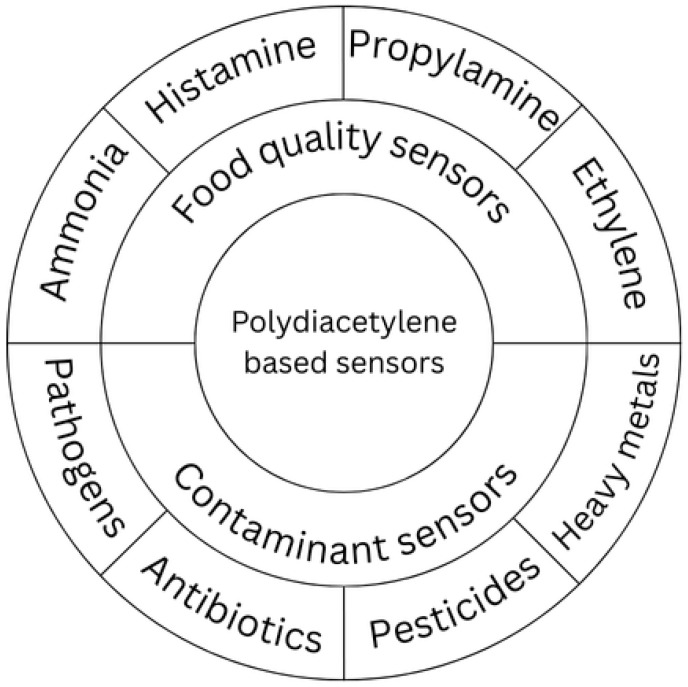
PDA sensor type and their target analytes.

These applications are summarized in Figure 3, which categorizes PDA-based sensors according to their role in quality assessment and contaminant detection.

### 4.1. Detection of Food Spoilage

Food products can spoil due to microbial growth, enzymatic activity, or chemical deterioration, leading to off-odors, off-flavors, and potential health hazards. Rapid detection of incipient spoilage is crucial to prevent distribution of unsafe foods and to reduce waste. Nanosensors offer powerful means to monitor the early signs of spoilage by targeting specific indicators. One major approach is the detection of spoilage microorganisms and their metabolites. Nanoscale biosensors have been developed to detect bacterial pathogens such as *Escherichia coli*, *Salmonella*, *Listeria*, and *Staphylococcus aureus* on foods much faster than traditional culture methods, often providing results within minutes to hours [43]. For instance, nano-biosensors employing antibodies or DNA probes on nanoparticle surfaces can capture bacteria from a food sample and produce an immediate signal (colorimetric, fluorescent, or electrochemical) proportional to the bacterial load. An example is the Food Sentinel System, which integrates antibody-functionalized nanoparticles into a barcode label: when pathogenic bacteria are present in the packaged food, they bind to the antibodies and cause a change in the barcode that can be optically detected, warning consumers and retailers [11]. Similarly, immunosensors using enzyme-linked nanoparticle assays have achieved sensitive detection of mycotoxins (toxic metabolites of fungi) like aflatoxins and ochratoxin in grains and nuts, which are common spoilage issues in storage [1].

Another set of spoilage indicators is chemical changes in the food’s environment. Spoilage microbes often produce volatile organic compounds (VOCs)—for example, anaerobic bacteria in meat release malodorous amines and sulfur compounds, and aerobic spoilage might increase carbon dioxide levels in a package. Nanosensors can be tailored to detect these spoilage gases or pH shifts that occur as food degrades. Colorimetric sensor strips containing pH-sensitive nanoparticle dyes or polymers are widely studied for this purpose: when the food’s headspace becomes acidic or alkaline beyond a threshold (due to microbial metabolites), the sensor strip changes color, providing a visual indication of spoilage [44]. For instance, nanocomposites of dyes with silica or polymer nanoparticles have been used to create freshness indicators in meat packaging that turn from green to red as meat spoils and pH drops. Similarly, sensors for total volatile basic nitrogen (TVB-N, a key metric of seafood spoilage) have been developed using graphene and metal-oxide nanomaterials that react with amine vapors and produce an electrical signal corresponding to fish freshness [45]. Electronic nose (e-nose) systems, which employ an array of nano-enabled gas sensors combined with pattern recognition algorithms, have shown success in evaluating the spoilage status of meats, fish, dairy, and produce by “smelling” the headspace gases [13,20]. For example, a set of nanostructured sensor films (one sensitive to ammonia, another to sulfides, etc.) can collectively classify beef or chicken samples as fresh or spoiled by the profile of gases detected—a task that traditionally would rely on human sensory evaluation or microbial plate counts.

Importantly, nano-sensing methods for spoilage are not limited to within-package monitoring. There are also portable nanosensor kits (akin to litmus paper or dipstick tests) that food inspectors or consumers can apply to a food item. One commercial example is a nanoparticle-based swab that, when rubbed on the surface of fresh produce or meat, will emit fluorescence if pathogenic bacteria are present [11]. Another example is a paper-based nanosensor for milk freshness: it contains immobilized enzymes and nanoparticles that react with increasing lactic acid (a product of milk souring) and produce a distinct color change visible to the naked eye. These on-demand tests, enabled by nanomaterials, empower quick decisions about product safety without the need for lab equipment.

The use of nanosensors for spoilage detection has significant implications: it allows for continuous or high-frequency monitoring rather than periodic sampling. An entire logistics chain can be surveilled by intelligent packaging that constantly “checks” the product status. If at any point the nanosensor signals danger (e.g., detection of threshold levels of bacterial contamination or toxin production), the product can be flagged and removed before reaching consumers [11]. This not only protects public health—given that hundreds of millions of illness cases annually are attributed to consumption of spoiled or contaminated foods [20]—but also helps reduce unnecessary waste by avoiding premature disposal. Foods that are still good will not be thrown out just based on conservative expiration dates, because real-time sensor feedback gives a true measure of quality [20]. Thus, nanosensors for spoilage detection support both food safety and sustainability.

Representative recent devices and their analytical performance are summarized in Table 4.

Label-type hydrogels and hybrid e-noses target decision-critical TVB-N/amine bands on the timescale of sub-minute to few-minute response times, yet matrix humidity and batch-to-batch dye variability remain operational constraints; array-based e-noses preclude false positives at the cost of model maintenance (drift compensation, recalibration).

Author Perspective. There is a pragmatic compromise between single-marker (e.g., xanthine, TVBN) and pattern-based e-nose approaches: single-analyte sensors are easier to calibrate to thresholds, but multi-sensor arrays capture a collective spoilage signature that is typically stronger across products and storage histories. For rapid go/no-go readout during reception, low-cost colorimetric tags are attractive; for traceability and trend detection on the cold chain, instrumented electrochemical or chemiresistive readouts provide more information. Humidity, temperature variability, and matrix volatiles remain dominant confounders—simple sample-handling protocols (pre-equilibration, headspace normalization) and on-board drift correction may precondition performance without adding complexity at the user interface. Finally, determining time-to-decision and false-alarm tolerance during initial design leads to better sensor-format selection and fewer field failures.

### 4.2. Detection of Intentional Food Tampering

Intentional food tampering—the illicit addition of inferior or harmful substances to foods, or misrepresentation of ingredients—is a longstanding concern in the industry. Common examples include dyeing of spices with industrial chemicals, dilution of high-value products (like olive oil or honey) with cheaper substitutes, or addition of melamine to fraudulently boost apparent protein content in milk. Nanosensors have shown great promise in detecting such adulterants quickly and reliably, even when present at very low concentrations or hidden in complex food matrices [51].

Emerging form factors in smartphones and paper-based (µPAD) assays also have excellent potential for on-site adulterant detection. A smartphone Au@carbon-QD assay detects melamine in milk down to 3.6 nM through phone-camera readout [52], and paper-based, disposable µPAD formats provide low-cost workflows and simple colorimetric/electrochemical routes to dyes and small molecules in complex foods [2,25]. These formats build on the strengths of conventional Au/AgNP colorimetry by adding portability, simple image-based quantitation, and supply-chain deployability.

Beyond plasmonic colorimetry, electrochemical nanozyme sensors have emerged to quantify plant polyphenols and adulterants with ultralow LODs and intelligent signal processing. For example, Cheng et al. designed hierarchical all-carbon nanozyme architectures that enable intelligent rutin detection with a 42 pM limit of detection and a 0.1 nM–13 µM linear range, using in situ electroreduction assembly and machine learning–assisted readout—an approach readily extensible to phenolic adulterants in complex food matrices [21].

One notable case is the detection of melamine in dairy products. Melamine (a nitrogen-rich chemical) was illegally added to milk powder to falsify protein tests, leading to a global food safety scandal. Conventional methods (HPLC or mass spectrometry) could detect melamine but require laboratory facilities. Researchers developed simple colorimetric nanosensors using gold and silver nanoparticles to spot melamine on-site: in the presence of melamine, these nanoparticles aggregate due to melamine binding, causing a visible color change in the solution [53]. For instance, a gold nanoparticle suspension, normally red, will turn blue or purple when melamine above a few ppm is present because the particles clump together [51]. Such a test can be performed by merely mixing a milk sample with the nanoparticle reagent—a color change indicates intentional melamine addition. The sensitivity is remarkable: limits of detection around 0.5–1 ppb (parts per billion) melamine have been achieved with optimized AuNP or AgNP systems [54], well below regulatory safety limits. These assays are also rapid (minutes) and do not require expensive instrumentation, illustrating how nanotech makes adulterant testing more accessible.

Another common form of food tampering is the use of illegal dyes in foods (for example, Sudan I dye in chili powder or curry to enhance color). Nanosensors employing magnetic or graphene-modified electrodes have been created to detect these synthetic dyes electrochemically. In one report, an electrochemical sensor based on Fe_3_O_4_ magnetic nanoparticles on a graphene electrode could detect Sudan I dye in chili powder with a detection limit in the nanomolar range [16]. The magnetic nanoparticles increased the electrode’s surface area and preconcentrated the dye (via adsorption), thereby amplifying the current signal. This sensor allowed screening of spice samples on-site by simply placing a bit of spice extract on the test strip and measuring the electrical response with a handheld reader. Similarly, preservative adulterants like excess sulfites in sugar or wine have been detected using fluorescent carbon dot nanosensors that light up in the presence of those chemicals [16].

Product tampering often involves economically motivated substitution, such as diluting pure fruit juice with sugar syrup. Nanosensors can be tailored to catch such substitutions by targeting marker compounds. For example, a nano-biosensor for added cane sugar in orange juice was developed by coating an electrode with a graphene–polymer composite that could measure subtle changes in the juice’s electrochemical profile when extra sucrose was present [54]. In honey, where added corn syrup is a concern, nanopore-based sensors (with enzyme-functionalized nanoporous membranes) have been used to measure isotope ratios or specific carbohydrate signatures indicative of pure vs. adulterated honey. While these approaches venture into sophisticated analyses, the use of nanostructured sensing elements remains key for sensitivity.

It is also worth noting that some nanosensors designed for contaminant detection double as tests for food fraud. For instance, heavy metals or pesticide residues in food (which may occur due to fraudulent additives or accidental contamination) can be detected by nano-enabled sensors: gold nanoclusters that fluoresce when binding mercury, or nanostructured enzyme assays that change color in the presence of organophosphate pesticides. These help to ensure that if a food is illegally adulterated with a hazardous preservative or residue, it can be identified before reaching consumers [4,22].

In summary, nanosensors significantly strengthen our ability to detect a wide array of food adulterants. They provide on-site, quick diagnostics that can be implemented at points of purchase or processing, thus acting as a deterrent against food fraud. By catching intentional contamination early, food companies protect their brand integrity and regulators can enforce standards more effectively. The combination of high sensitivity (down to trace levels) and specificity of nanomaterial-based detection methods is a game-changer in assuring food authenticity and safety in global supply chains.

Selected nano-enabled sensors for adulteration control are collated in Table 5 with quantitative figures of merit for direct comparison.

In comparison with microfabricated electrochemical biosensors, nano-enabled platforms tend to achieve lower LODs and response times in food matrices via high-surface-area and catalytic/plasmonic amplification—albeit with potential vulnerability to signal drift and batch-to-batch nanomaterial variation, whereas microfabricated platforms excel in reproducibility and calibration transfer [39,59]. Compared to polymer-only indicators (e.g., PDA freshness tags), nanomaterial hybrids offer quantitation and multi-analyte selectivity (DPV/EIS/ratiometric) at the cost of added device complexity; PDA-only tags remain ultra-low-cost, eye-readable go/no-go indicators suitable to threshold decisions and consumer-friendly labels [22]. Practically, hybrid strategies—a polymer indicator for logistics triage with a nano-electrochemical confirmatory step—balance cost, ruggedness, and analytical confidence for shelf-life grading and adulterant screening [11].

Au/AgNP colorimetry achieves screen-level LODs lower than Codex melamine maxima with minimal hardware, whereas magneto-electrochemical formats (e.g., Fe_3_O_4_/EQCM) offer confirmatory-grade sensitivity but require an electrochemical readout; smartphone fluorescence (Au@CQDs) sacrifices field deployability in exchange for ppb-level performance.

### 4.3. Smart Packaging

“Smart packaging” refers to packaging systems that actively monitor or interact with the food and its environment to provide information on the product’s status and, in some cases, to improve its shelf-life. Nanotechnology has been a driving force behind recent improvements in smart packaging, enabling the development of thin, embedded sensors and active functionalities that were not feasible with traditional materials [11,20]. There are generally two aspects to smart packaging: (1) indication/communication, where the package senses change and signals information (e.g., a color change indicating spoilage, or an RFID sensor transmitting data), and (2) intervention, where the package actively controls or adjusts conditions (e.g., releasing an antimicrobial agent). Many nano-enabled packages combine both, effectively becoming an intelligent micro-environment for the food.

One of the most straightforward smart packaging innovations is the use of nanomaterial-based indicator labels. These are small inserts or printed patches inside packaging that change their optical properties in response to certain conditions. For example, a popular concept is a time–temperature indicator (TTI) that records the thermal history of the product: if a refrigerated product has been exposed to abuse (higher temperatures) for too long, the TTI will undergo a chemical reaction that causes a visible color change. Nanoengineered TTIs use encapsulated dyes or polymer nanoparticles that gradually react or diffuse at a rate dependent on temperature, providing an integrated record of the product’s exposure over time [11]. Similarly, freshness indicators using nanomaterial-embedded dyes can respond to spoilage volatiles. For instance, a label containing PDA (polydiacetylene) vesicles might remain blue as long as the food is fresh but turn red once amines from protein decomposition build up beyond a threshold [22]. Such colorimetric signals are eye-readable, allowing consumers or inspectors to judge quality immediately without instruments [22]. This concept parallels the “eye-readable sensors” used in other fields (for example, gasochromic films that visibly indicate hydrogen leaks) [60], repurposed here for food gases like carbon dioxide or ammonia in a package.

In smart packaging, nanosensors can also be coupled with electronic communication devices. Several recent packaging prototypes include printed nanosensor circuits—essentially, small sensor tags containing nanomaterial-based sensing elements and a wireless communication chip (such as RFID or NFC). These tags can be very thin and flexible, even printed with conductive nano-inks, and can be attached to individual packages. They might monitor temperature, humidity, or gas composition inside the package and then transmit that data when scanned by a reader or smartphone. For example, a milk carton could have an RFID tag with a nano-infused chemical sensor that tracks pH; a grocery store employee can scan the carton and the tag will send a signal if the pH indicates spoilage. The integration of smartphone-readable sensors is an especially promising development—consumers could simply tap the package with their NFC-enabled phone and receive a real-time readout of freshness [20]. Research has demonstrated such systems using carbon nanotube-based gas sensors and miniaturized electronics, effectively creating an “Internet of Food” where each item can report its condition.

On the active packaging side, nanomaterials improve packaging functionality in several ways. Antimicrobial nano-coatings are one prominent application: by embedding nanoparticles like silver, ZnO, or naturally derived nanoscale antimicrobials (e.g., nano-chitosan) into packaging films, the material gains the ability to suppress microbial growth on the food surface [17,18]. This not only preserves the food but also complements the sensing aspect—the packaging can both detect bacteria and help eliminate them. Some smart films are being designed to release antimicrobial agents in a controlled manner when sensing a microbial population increase. For instance, a film might contain nanoencapsulated essential oils that are released only when the pH drops (signaling bacterial fermentation) [11]. This kind of feedback-responsive packaging relies on nanomaterial carriers to act at the right time, extending shelf-life intelligently rather than continuously.

Another function is oxygen scavenging: nanomaterials like nano-iron or TiO_2_ can consume oxygen inside a package (through oxidation reactions) to maintain anoxic conditions and thereby slow aerobic spoilage [36]. While oxygen scavengers have existed before, nanotechnology makes them more efficient and easier to integrate as coatings or inserts. We can consider these as part of smart packaging, since they respond to and modulate the package atmosphere.

Nanoclay-based films also contribute to smart packaging by maintaining modified atmospheres. For example, a nanocomposite film with intercalated clay has a much lower oxygen transmission rate, which helps keep oxygen-sensitive foods (like fresh produce or roasted coffee) fresher for longer [61,62]. Though passive, this enhanced barrier is “smart” in the sense of preserving quality.

Interactive smart labels using QR codes or barcodes combined with nanosensors are an emerging idea: the barcode is printed with special nanoink that changes when the product is spoiled (becoming unscannable or altering the encoded data). The consumer or retailer scanning the code would automatically receive an alert if the code has changed due to product spoilage [11]. One example is a concept where a barcode printed on a perishable item becomes blurry if the item spoils—this was achieved by embedding an immunochemical sensor in the barcode that reacts with bacterial antigens, distorting the printed pattern upon reaction [11].

Smart packaging is especially valuable for cold chain management. With nano-enabled sensors tracking temperature or spoilage continuously, stakeholders can identify breaks in the cold chain (e.g., a pallet of yogurt left too long on a loading dock) and make informed decisions (perhaps rerouting it for immediate sale or disposal). This real-time data helps reduce both health risks and unnecessary waste of foods that might otherwise be discarded out of caution rather than actual spoilage. Indeed, studies show that a huge fraction of food waste comes from uncertainty about product freshness [20]. By providing dynamic shelf-life information—that is, a shelf-life that updates based on actual storage conditions rather than a fixed date—smart packaging can ensure that foods are used until truly no longer good, and that unsafe foods are detected even if issues occur before the printed expiration [20].

In conclusion, nanomaterials play an integral role in the latest smart packaging technologies. Whether as part of a sensor that informs (colorimetric labels, RFID nanosensors) or as part of a response system that protects (antimicrobial and barrier nanocomposites), they contribute to packaging that is more than a passive container. The result is a shift toward intelligent food packaging that can communicate with consumers and supply chain managers, improve safety, and lengthen shelf-life. This shift is well underway, with commercial implementations beginning to appear (e.g., antimicrobial nano-packaging for meat, produce stickers that indicate ripeness). Continued advances in printable electronics, low-cost nanosensor fabrication, and food-safe nanomaterials will further accelerate the adoption of smart packaging in the coming years.

Author Perspective: In smart packaging, the best approach is to enhance the passive barrier (nanoclays, silica, nanocellulose) first and only secondarily incorporate indicators or active functions when required; this sequential development tends to give maximum shelf-life gain per unit cost and minimizes migration risks. Hybrid structures with a simple indicator (pH/VOC or freshness mark) plus a defined active function (antimicrobial or oxygen scavenger) comprise an even keel approach to scaling up and consumer trust. Materials and manufacturing should be chosen with printing/lamination compatibility and end-of-life in mind (recyclability or compostability), as regulatory approval more and more weighs life cycle against toxicology. Lastly, with wireless tags, prioritize reader interoperability (RFID/NFC) and data minimization to avoid putting loads on retailers’ infrastructure.

### 4.4. Real-Time Monitoring

One of the most transformative aspects of applying nanotechnology to food sensory systems is the ability to achieve real-time, continuous monitoring of food conditions. Traditional quality testing often occurs at discrete points (for example, testing a sample from a batch in a lab). In contrast, nanotechnology enables the deployment of numerous tiny sensors throughout the food production and supply chain, which can continuously or periodically collect data and even communicate it wirelessly. Real-time monitoring means that at any given moment, the status of a food product (whether it is still fresh, if it has been exposed to temperature abuse, if contamination has occurred, etc.) can be known and appropriately acted upon.

In practical terms, many of the smart packaging examples discussed already serve the purpose of real-time monitoring. For instance, a nanosensor embedded in a package that continuously fluoresces in proportion to bacterial growth is effectively monitoring in real time; a data logger that records the output of a nano-based TTI or gas sensor over the course of distribution provides a real-time (or near-real-time) history. What distinguishes this section is the emphasis on the systems aspect—linking sensors into networks and ensuring continuous data flow. Nanomaterials contribute by making sensors small and power-efficient enough to be ubiquitously placed on products.

An emerging concept is the “Internet of Things” (IoT) for food, sometimes dubbed the Internet of Nano-Things when nano-sensors are used. In this framework, each food package or storage unit can be equipped with a tag containing one or more nanosensors and a wireless transmitter. These tags can send data to a cloud-based system at set intervals. For example, a warehouse might have thousands of milk cartons, each with a nanostructured freshness sensor and an RFID/NFC chip; as they sit in storage, readers pick up signals from each carton. If one carton shows a spike in, say, bacterial metabolites (perhaps due to a sealing defect in that package alone), the system can immediately flag that specific item for removal, rather than discovering the spoilage at the retail shelf or by consumer complaint. Integrating nanosensor data with digital networks allows pinpoint control and traceability. Manufacturers can monitor products through transit—if a refrigeration unit fails during trucking, the nanosenors will catch temperature rises or early spoilage signs en route, and the affected batch can be diverted or inspected on arrival.

Some pilot studies have demonstrated such systems: one example used radio-frequency temperature sensors made with nanoparticle-printed antennas on fruit shipments; the sensors continuously recorded the temperature, and the data was transmitted when the truck came within range of a receiver at its destination [39]. In another case, researchers placed oxygen and humidity nanosensors in silos of stored grain and monitored the conditions remotely, enabling early detection of moisture ingress that could lead to mold growth. These scenarios show how continuous monitoring can prevent large-scale spoilage by addressing issues at inception.

For real-time monitoring to be truly effective, the sensor’s response must be rapid and preferably reversible or renewable. Many nanomaterial sensors respond almost instantaneously to changes (e.g., a metal-oxide gas sensor’s resistance changes within seconds of a gas’ appearance). Some sensors, like colorimetric labels, may have a one-time irreversible change (they are meant to record an event). Others, however, can be designed to fluctuate with the environment; for instance, a photonic crystal sensor might change color when CO_2_ levels rise and revert if CO_2_ decreases. These reversible nanosensors can therefore track conditions dynamically. A practical example is a freshness sensor for fish that uses a film of pH-responsive polymer nanofibers: as fish spoils, it releases basic amines, and the film’s color shifts from yellow to green; if by some intervention the amines are reduced (not typical in fish, but conceptually), it would shift back. During normal progression, the color intensifies over time, and readings (obtained via a smartphone app that quantifies color) taken every few hours show a curve of increasing spoilage, essentially providing a timeline of freshness degradation.

Another fascinating frontier is combining nanosensors with active control systems for real-time quality management. Imagine a storage chamber that has an array of nanosensors and devices that can modulate conditions (like fans, filters, and UV lights). If the sensors detect a spike in ethylene (signaling fruit ripening), the system could vent the ethylene or cool the chamber to slow the ripening. If humidity rises in a salad bag (risking condensation and decay), a moisture-absorbing nanomaterial could be activated. This kind of closed-loop control, guided by continuous sensor feedback, is akin to how a thermostat regulates home temperature, but here regulating a microclimate around a food product.

Real-time monitoring, especially when scaled to an entire supply network, generates a vast amount of data. This is spurring interest in data analytics and machine learning applied to food quality data. With consistent input from nano-sensors, algorithms can learn patterns—for example, correlating certain temperature fluctuation patterns with shelf-life reduction. Over time, this could enable predictive models that help producers optimize distribution (rerouting products predicted to spoil sooner to nearer markets, etc.). Essentially, the nano-sensors provide the rich data stream needed for an increasingly “smart” supply chain that can make autonomous decisions to maintain quality.

From the consumer’s perspective, real-time monitoring ensures that by the time a food item is purchased, its history is known, and it can be trusted. Some companies have started to include scannable sensor-based freshness labels on high-value perishable foods like seafood; a customer can scan the package at point of sale and see a simple indicator of whether it is still within peak freshness. This level of transparency is new, and it relies on continuous monitoring from packing to retail.

**Figure 4:**
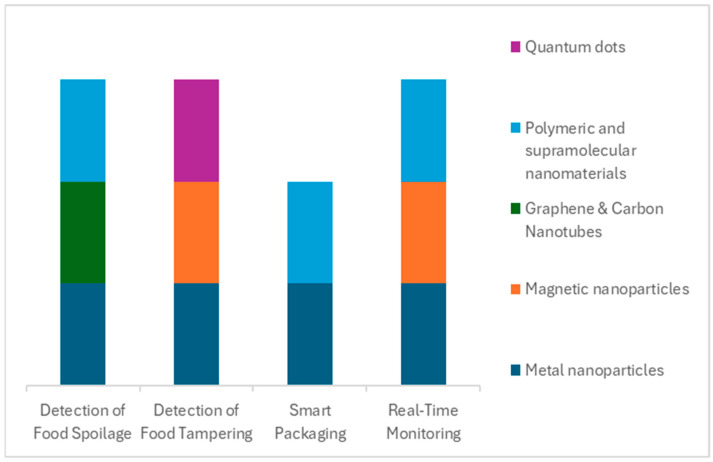
Correlation between nanomaterial type and areas of application.

In summary, nanotechnology not only creates sensitive sensors but also facilitates a paradigm shift from periodic testing to continuous monitoring in food systems. The combination of nanosensors, wireless communication, and data systems means food quality can be tracked in real-time, much like vital signs in a patient. This greatly improves responsiveness—potential problems can be addressed as they happen—and thereby enhances both food safety (reducing risk of outbreaks and recalls by catching issues early) and quality management (ensuring consumers get the freshest possible products). Real-time monitoring is still in a growing phase, with pilot implementations in logistics and inventory management, but it is expected to expand as sensor costs drop, and the food industry adopts more IoT infrastructure. Nanomaterials will be central in this expansion, as they offer the performance needed for reliable and widespread sensing, see Figure 4.

## 5. Advantages of Nanomaterials

Nanomaterials’ incorporation into food sensing systems yields quantifiable performance gains due to high surface-to-volume ratios and surface/optical/electronic tailoring. The most consequential outcomes in food analysis are trace-level detection, discrimination among multicomponent mixtures, fast kinetics suitable for online/real-time use, and the potential for sensing to be integrated with packaging protection functionalities.

- **High Sensitivity and Low Detection Limits**: Perhaps the most celebrated advantage of nanosensors is their extraordinary sensitivity. Because nanoscale sensors operate at a length scale comparable to target analytes (molecules, bacteria, etc.), they can interact with targets very efficiently. A nanosensor’s surface can be functionalized with a dense layer of bioreceptors (e.g., thousands of antibodies on a single nanoparticle), boosting the probability of capture of the analyte. Moreover, many nanomaterials (like metal nanoparticles or quantum dots) exhibit signal amplification phenomena—for instance, a small amount of analyte can induce aggregation of many nanoparticles, producing a strong color change. Compared to traditional microscale sensors, nanoengineered materials can recognize distinct analytes present at extremely low concentrations [2]. In practical terms, this means detecting contamination or spoilage markers earlier, when their levels are still minimal. For example, nanocomposite sensors have detected pathogens at <10^2^ CFU/mL and toxins in the ppb range, thresholds that were very challenging to reach with older technology. The high surface area of nanomaterials leads to larger signal outputs (e.g., bigger changes in conductivity or more fluorescence) per unit of analyte captured [1,2]. Enhanced sensitivity is crucial for food safety, as it enables early warning—nanosensors might detect the onset of contamination before it spreads or becomes dangerous.
- **Greater Specificity and Selectivity:** Through surface engineering, nanomaterials can be made highly selective to the target of interest despite complex food matrices. For instance, a nanoparticle can be coated with molecularly imprinted polymers that only bind a specific antibiotic residue, or a carbon nanotube transistor can be functionalized with DNA that hybridizes only with a pathogen’s genome. The result is that nanosensors can often distinguish the target from a background of other substances (minimizing false positives/negatives). Additionally, certain nanomaterials inherently provide selectivity, e.g., enzyme-mimicking nanocatalysts might only catalyze a reaction with a particular sugar, or a gold nanosensor might only change color with molecules that induce a certain inter-particle interaction. In a field like food, where samples are complex (containing fats, proteins, etc.), having high selectivity is as important as sensitivity. Nanotechnology has facilitated creating “lock-and-key” recognition at the nanoscale. This selectivity is evident in sensors like aptamer-conjugated gold nanoparticles that only aggregate in the presence of the aptamer’s target (say, aflatoxin B1), producing a color change only for that toxin and not for others [40]. Traditional methods struggled with matrix interferences, but nanomaterials with tailored surface chemistry help sidestep many cross-reactivity issues.
- **Rapid Response and Real-Time Detection:** Nanosensors often exhibit very fast response kinetics. Due to their small size, they have short diffusion distances for analytes and can quickly reach equilibrium. Electrical nanosensors (like nanowire or nanotube sensors) respond essentially in real time as target molecules adsorb—changes in current or potential occur within seconds. This means that nanotechnology-based tests can be much quicker; for example, a nanoparticle immunosensor might provide a result in a few minutes whereas an ELISA assay takes hours. This rapidity is vital for applications like checking raw milk for antibiotics at the collection center or screening meat for pathogens during processing, enabling near-instant decisions. Some reports show detection of toxins or spoilage gases in under one minute using nanoengineered electrodes, which is orders of magnitude faster than sending a sample to a lab [11]. The ability of certain nanosensors to operate continuously (as discussed in real-time monitoring) further underscores their advantage in providing immediate feedback.
- **Integration of Sensing and Preservation:** A unique advantage in food applications is that nanomaterials can simultaneously serve as sensors and active protectants. This dual functionality is rarely possible with macroscale materials. For instance, silver nanoparticles in a film can, on one hand, act as an antimicrobial to kill bacteria (preservative function), and on the other hand, changes in their optical properties can indicate the onset of bacterial growth (sensor function) by, say, a plasmon resonance shift. A single nanocomposite coating might scavenge oxygen (prolonging shelf-life) and change color when the oxygen-scavenging capacity is exhausted (signaling package integrity failure). Thus, nanotechnology allows a convergence of roles—packaging that is both smart and active. This can simplify designs and reduce costs, because one component fulfills multiple purposes. Enhanced food safety is achieved not just by detecting a problem but by mitigating it, e.g., a nanofiber mat in a fish package might release antimicrobial peptides when the temperature rises (an active role) and contain a thermochromic nanopigment that records that temperature excursion (an indicator role). Traditional packaging would need separate elements for each role. By integrating functions, nanomaterials help maintain food quality while monitoring it.
- **Improved Packaging Material Properties:** Aside from direct sensing, nanomaterials improve the mechanical and barrier properties of packaging, which is an indirect advantage for food quality. Nanofillers like nanoclays, nano-cellulose, or silica nanoparticles reinforce polymers, making them stronger, more puncture-resistant, and sometimes more flexible [17].
- **Enhanced Sensitivity and Selectivity**: One of the most significant advantages of nanomaterials in sensory systems is their ability to detect extremely low concentrations of analytes with high specificity. The high surface-area-to-volume ratio of nanoparticles allows for increased interaction with target molecules, improving the detection of pathogens, toxins, and spoilage indicators [1].

For example, gold nanoparticles exhibit localized surface plasmon resonance (LSPR), a property that changes their optical behavior when they interact with specific substances. This feature enables the creation of highly responsive colorimetric sensors that can visually indicate the presence of contaminants such as pesticides, toxins, or adulterants.

Electrochemical sensors constructed from carbon nanotubes (CNTs) or graphene exhibit ultra-fast electron transfer and low background noise, enabling the real-time monitoring of analytes such as ammonia, ethanol, and hydrogen sulfide [17,18]. This sensitivity not only ensures food safety by detecting contaminants early but also helps monitor product quality dynamically throughout the supply chain.

- **Antimicrobial and Antioxidant Properties:** Many nanomaterials inherently possess antimicrobial or antioxidant functions that enhance the protective features of food packaging or detection systems. Silver nanoparticles (AgNPs), for example, are widely known for their broad-spectrum antimicrobial activity, which is attributed to their ability to disrupt bacterial cell membranes and generate reactive oxygen species. Zinc oxide (ZnO) and titanium dioxide (TiO_2_) nanoparticles also exhibit antimicrobial effects, particularly when activated by light.

This dual function of detection and preservation is particularly valuable in perishable products like dairy, seafood, and ready-to-eat meals, where microbial growth and oxidative degradation are primary concerns. Moreover, the sustained antimicrobial activity of nanomaterials like silver nanoparticles contributes to extended shelf-life without the need for synthetic preservatives.

- **Improved Nutrient Delivery and Functionalization:** In addition to detection and preservation, nanomaterials such as nanoliposomes and nanoemulsions offer benefits related to nutrient delivery and functional food formulation. These systems encapsulate bioactive compounds like vitamins, antioxidants, polyphenols, or omega-3 fatty acids, protecting them from degradation during storage and digestion. Nanoemulsions, with their small droplet size and high kinetic stability, serve as ideal carriers for fat-soluble vitamins and antioxidants in functional foods and nutraceuticals. These systems enhance shelf stability and enable release triggered by physiological conditions like pH or enzymes [2].
- **Flexibility in Sensor Design and Integration:** Nanomaterials can be easily integrated into a wide range of platforms—from paper-based sensors and flexible films to RFID tags and smartphone-compatible devices. This flexibility supports innovation in packaging, retail, and even consumer-use applications. For example, graphene-based materials can be printed or coated onto plastic substrates to create thin, transparent sensors for freshness detection. Such sensors can be integrated into food labels, smartphone-connected devices, or interactive packaging, enabling real-time feedback for producers and consumers alike [24].
- **Environmental and Economic Efficiency:** From a sustainability perspective, nanomaterials enable more efficient food monitoring and packaging strategies. Their multifunctionality allows for the reduction of material usage, replacement of multiple additives, and minimization of food waste. Smart sensors embedded in packaging can prevent premature disposal of safe food by providing objective freshness data, supporting circular economy principles. Economically, nanomaterials are becoming more accessible due to advances in synthesis methods and scalable production. The resulting reduction in sensor costs makes them viable not only for industrial applications but also for consumer-level use, such as in-home freshness indicators or portable test kits, see Table 6.

## 6. Challenges and Future Trends

While nanomaterials present transformative potential in food sensory systems, their application is not without significant challenges. From concerns about toxicity and human health to regulatory ambiguity and issues related to scalability, the implementation of nanotechnology in the food sector must navigate a complex landscape. This chapter explores the primary obstacles that researchers, manufacturers, and policymakers must address to ensure the safe and effective integration of nanomaterials in the food industry.

- **Toxicological Concerns and Human Health**: Perhaps the most pressing challenge in applying nanomaterials to food systems is their potential toxicity. Due to their nanoscale dimensions, these materials can cross biological barriers, interact with cellular components, and accumulate in organs. Ingested nanoparticles may translocate across the intestinal lining and enter systemic circulation, raising concerns about long-term exposure and bioaccumulation.

The toxicity of nanomaterials depends on multiple factors, including size, shape, surface charge, solubility, and chemical composition. For example, silver nanoparticles—which are widely used for their antimicrobial activity—have been shown in some studies to cause oxidative stress, inflammation, and DNA damage in mammalian cells [59]. Similarly, zinc oxide and titanium dioxide nanoparticles, though generally recognized as safe (GRAS) in certain concentrations, may exert cytotoxic effects at higher doses or under specific environmental conditions [60].

One of the key difficulties in evaluating nanomaterial toxicity is the lack of standardized testing protocols and dose–response metrics tailored for nanoscale substances. Many existing toxicological assessments were developed for bulk materials and may not capture the complex interactions unique to nanomaterials [22].

Safe-by-design for carbon nanomaterials and quantum dots: Practical mitigation combines surface passivation (e.g., PEG/PVP) and biopolymer encapsulation/coatings (chitosan, cellulose) to curb ion release and ROS while retaining optical yield [12]; barrier-first layouts (oxide/clay layers) and low-migration architectures (immobilization in cross-linked matrices) are preferred for in-package uses [17,18]. Whenever feasible, green synthesis routes (plant/microbial) and benign solvents should be adopted to align with food-contact expectations [35]. From a governance standpoint, food-additive safety principles and scoping evidence on human/environmental impacts argue for testing on the final article (not free nanoparticles), migration studies under worst-case conditions, and transparent reporting [63,64].

- **Environmental Impact and Bioaccumulation**: In addition to human health, environmental risks posed by nanomaterials are a growing concern. Once released into wastewater, soil, or the atmosphere, nanoparticles can interact with plants, microbes, and aquatic organisms. Their persistence and mobility in ecosystems raise questions about their long-term ecological footprint.

Studies have indicated that certain nanoparticles can inhibit plant growth, disrupt microbial communities, and accumulate in aquatic organisms, potentially entering the food chain [2]. These unintended consequences underscore the need for life cycle assessments and eco-toxicological evaluations when developing and deploying nanotechnology-based food products.

- **Regulatory and Legal Uncertainty**: The regulatory environment for nanomaterials in food applications remains fragmented and underdeveloped. While agencies such as the U.S. Food and Drug Administration (FDA) and the European Food Safety Authority (EFSA) have issued guidance documents, there is no unified global framework governing the approval, labeling, and monitoring of nano-enabled food products [24].

A major challenge lies in the definition and classification of nanomaterials. For instance, some regulations define a nanomaterial based strictly on particle size, while others consider functionality or manufacturing method. This inconsistency complicates compliance and enforcement efforts.

Moreover, there is a lack of transparency in commercial formulations that include nanomaterials. In many cases, manufacturers are not required to disclose nano-ingredients on product labels, limiting consumer awareness and informed decision-making. This lack of clarity has fueled public skepticism and reduced acceptance of nanotechnology in food [12].

- **Scalability and Economic Viability**: While laboratory-scale studies on nanomaterial-based sensors and packaging are promising, transitioning to commercial production presents several logistical and economic challenges. Scaling up synthesis processes while maintaining uniformity, safety, and cost-efficiency is complex.

Batch-to-batch variability in nanoparticle size or surface characteristics can significantly affect sensor performance and safety profiles. Moreover, high-purity nanomaterials often require sophisticated equipment and specialized handling, which can increase production costs and limit adoption by smaller manufacturers [58].

- **Public Perception and Ethical Issues**: Consumer acceptance of nanotechnology in food remains mixed, and is largely influenced by perceptions of risk, media narratives, and trust in regulatory institutions. Surveys suggest that while consumers may welcome improved food safety and shelf-life, they are wary of ingesting particles they cannot see or understand [20].

Ethical considerations also arise regarding the equitable distribution of nano-enabled food technologies. High production costs may restrict access to wealthier regions, potentially exacerbating global disparities in food quality and safety [64].

- **Next-Generation Smart Packaging**: One of the most promising frontiers in food nanotechnology lies in smart packaging systems capable of multifunctional sensing and adaptive response. Future packaging materials are expected to incorporate networks of nanosensors that monitor multiple parameters simultaneously—such as gas composition, humidity, temperature, and microbial contamination—allowing comprehensive real-time assessment of food status [1]. These systems may also be equipped with actuators that release preservatives or antimicrobial agents in response to spoilage signals, creating dynamic packaging solutions that not only detect but also mitigate deterioration.

Emerging materials such as cellulose nanofibers, biodegradable polymers, and edible nanocomposites are expected to replace synthetic substrates in packaging, aligning with circular economy principles. Combining these with functional nanomaterials like ZnO or graphene oxide will enable low-cost, sustainable sensors that are both scalable and consumer-safe [18].

- **Integration with Digital Platforms and IoT**: The convergence of nanotechnology with the Internet of Things (IoT), artificial intelligence (AI), and cloud computing will redefine how data from nanosensors is collected, analyzed, and acted upon. Wireless nanosensors embedded in packaging or along the supply chain can relay freshness data to cloud-based systems where algorithms predict spoilage trends and optimize logistics [4].

Smartphone-linked nanosensors—already demonstrated in lab-scale systems—will soon be refined for commercial use. Consumers may be able to scan QR codes to access real-time freshness, storage, and safety data measured directly by embedded nanosensors [22]. This transparency will increase consumer trust while reducing unnecessary food waste, see Table 7.

Despite the immense promise of nanomaterials in food sensory systems, several challenges must be addressed to ensure their safe, sustainable, and socially accepted application. Toxicological risks, environmental impact, regulatory gaps, and public perception issues form a complex matrix that requires coordinated efforts across disciplines. By proactively identifying and mitigating these barriers, stakeholders can unlock the full potential of nanotechnology in transforming food systems.

**Author Perspective**: A reliable path to deployment entails low-migration designs (encapsulation of nanomaterials in stable matrices) and toxicology on the ultimate article, not free nanoparticles. Validation panels based on standardized criteria—stress tests over temperature/humidity and realistic interference—should accompany field readiness claims, with results being compared against workhorse non-nano comparators to demonstrate actual value added. As increases in linked packages translate into data, governance (ownership, privacy, and audit trails) will be just as significant as sensor accuracy. The bulk of future rapid progress will come from modular platforms (interchangeable electronics + single-use nano-sensing inserts) and open reporting of adverse results, enabling reproducibility and fair comparison between studies.

## 7. Conclusions

The integration of nanomaterials into food sensory systems represents a remarkable leap toward smarter, safer, and more responsive food quality monitoring. Through this review, we have examined how various nanomaterials—ranging from metal and carbon-based structures to nanoceramics and nanoemulsions—can offer enhanced sensitivity, selectivity, and multifunctionality in detection systems.

Each category of nanomaterials contributes distinct advantages. Metal and magnetic nanoparticles provide powerful antimicrobial and optical detection capabilities. Carbon nanostructures add electrical sensitivity and adaptability. Nanoceramics bring photocatalytic and barrier-enhancing features, while nanoemulsions and nanoliposomes offer effective delivery and encapsulation mechanisms.

These technologies, when incorporated into smart packaging or continuous monitoring systems, enable real-time feedback on product status, enhancing traceability across the supply chain. Moreover, their potential to reduce waste, extend shelf-life, and support sustainable packaging aligns well with both industry goals and environmental responsibility.

Despite the progress that has been made, important considerations remain. Safety concerns regarding human exposure and environmental accumulation must be addressed through robust testing and transparent communication. The success of nanotechnology in the food industry will depend on responsible development, public acceptance, and harmonized regulatory frameworks.

Overall, nanomaterials are set to play an increasingly central role in modern food systems. With interdisciplinary collaboration and a focus on ethical, scalable, and sustainable solutions, these technologies have the capacity to transform how we monitor, protect, and trust our food.

## Figures and Tables

**Table 1:** Overview of major nanomaterial classes used in food-industry sensory systems, typical detection modes, and representative applications.

Nanomaterial Type	Detection Mode(s)	Representative Applications	References
Metal nanoparticles (Au, Ag)	Optical (plasmonic/colorimetric); electrochemical (NP-modified electrodes); SERS	Melamine detection in milk via nanoparticle aggregation (red→blue color shift); colorimetric sensing of small-molecule adulterants; sucrose on AuNP-modified electrodes;	[14,15]
Magnetic nanoparticles (Fe_3_O_4_, magnetic ferrites)	Immunomagnetic separation (IMS); electrochemical; magneto-piezoelectric (EQCM)	Pathogen capture and pre-enrichment for rapid readout; Sudan I dye detection on Fe_3_O_4_-modified electrodes; magnetic nanolabels boost EQCM/electrochemically coupled mass signals.	[5,16]
Metal oxide and ceramic materials (ZnO, TiO_2_, SiO_2_, nanoclays)	Chemiresistive; photocatalytic/antimicrobial (UV-activated); photoelectrochemical;	Detection of TVB-N/amine vapors; antimicrobial/photocatalytic active films; improved O_2_/H_2_O barrier in smart packaging; MOx and 2D semiconductors are frequently used in PEC food sensors.	[8,17,18]
Carbon-based (CNTs, graphene, carbon quantum dots; all-carbon nanozymes)	Electrochemical; chemiresistive; fluorescent/photonic	E-nose (spoilage VOC profiles); electrochemical detection of xanthine/amines (fish freshness); fluorescent sensing (CQDs). Nanozyme example: intelligent rutin sensing (model for phenolic adulterants)	[19,20,21]
Functional polymers (PDA, MIPs)	Colorimetric (PDA); electrochemical/impedimetric (MIP)	Freshness/amine indicators (PDA); pesticide screening (e.g., PDA + AgNP composites); MIP-based recognition of dyes/antibiotics	[22,23]
Nanoencapsulation systems (nanoemulsions, nanoliposomes, biopolymer nanocarriers)	Indicator/active release (triggered); supports smart packaging functions	Controlled release of antimicrobials/antioxidants in active films; responsive labels delivering on-cue signals	[13,24]

**Table 2:** Signal-transduction mechanisms by nanomaterial class (concise primer).

Nanomaterial Class	Representative Examples	Main Sensing Mechanisms/Functionalities
Noble-metal nanoparticles	Au, Ag	Optical: localized surface plasmon resonance (LSPR) peak shift or ratio change upon aggregation/refractive-index variation. Catalytic: peroxidase-like nanozyme activity for colorimetric or electrochemical detection. Electrochemical: redox mediation and surface-confined electron transfer at NP-modified electrodes.
Magnetic iron oxides	Fe_3_O_4_, ferrites	Electrochemical detection at Fe_3_O_4_-modified electrodes (e.g., dyes, adulterants). Immunomagnetic capture and pre-concentration enhancing amperometric/impedimetric signals. Magneto-piezoelectric (EQCM) labeling.
Metal oxides and ceramics	ZnO, TiO_2_, SiO_2_, nanoclays	Chemiresistive sensing of VOCs or biogenic amines. Photoelectrochemical enhancement under illumination. Antimicrobial and barrier roles in smart packaging.
Carbon nanomaterials	Graphene, CNTs, carbon dots	Electrochemical transduction (DPV, EIS, chronoamperometry) on high-surface conductive scaffolds. Chemiresistive or FET-based responses. Fluorescence quenching and recovery with carbon dots.
Quantum dots	II–VI QDs, carbon QDs	Fluorescent or ratiometric probes for ions and small molecules. Smartphone-readable formats.
Functional polymers	Polydopamine (PDA), MIPs, dendrimers	Colorimetric PDA blue→red transition (amines or pH). Electrochemical/impedimetric readout using molecularly imprinted polymer (MIP) layers.

**Table 3:** Comparative evaluation of methods.

Method	Examples	Advantages	Limitations	Relevance to Food Industry
Mechanical milling	Ball milling of metals/oxides	Simple, cost-effective	Broad size distribution, structural defects	Limited
Lithography	Nanoimprint, e-beam	High precision	Expensive, complex	Minimal
Laser ablation	Ag, Au nanoparticles	Pure, chemical-free nanoparticles	High energy cost	Moderate
Chemical reduction	Ag, Au, Cu nanoparticles	Scalable, antimicrobial activity	Toxic reducing agents possible	High (packaging, sensors)
Sol–gel process	TiO_2_, SiO_2_	Controlled porosity, stability	Slow, sometimes uses solvents	High (coatings, sensors)
Hydrothermal synthesis	ZnO, Fe_3_O_4_	Shape and size control	Requires pressure vessels	High
Green synthesis	Plant/microbial extracts	Eco-friendly, biocompatible	Slower, less precise	Very high (food-compatible)
Biological synthesis	Bacteria, fungi, enzymes	Sustainable, functionalized nanoparticles	Scale-up challenges	High
PVD/CVD	Graphene, CNTs, metal oxides	Uniform coatings, advanced materials	Equipment cost	High (smart packaging, sensors)

**Table 4:** Representative nano-enabled freshness/spoilage sensors (quantitative examples).

Target (Matrix)	Nanomaterial and Transduction	Performance (LOD/Range/Time)	Notes	References
TVB-N/ammonia (fish/meat)	Agarose hydrogel dye label (juglone); colorimetric	Detects 0.05 mg L^−1^ NH_3_ in ~8 min	Smartphone-friendly RGB analysis; on-package label	[46]
TVB-N (fish; smartphone)	Au@MnO_2_ nanozyme hydrogel; smartphone RGB	Strong RGB-TVB-N correlation; real-time monitoring	Smartphone-integrated hydrogel indicator	[47]
Biogenic amines (meat)	PDA hydrogel beads; colorimetric	Fast visible blue→red transition; (semi-quant.)	Simple, low-cost spoilage indicator	[41]
Trimethylamine (salmon label; smartphone)	Colorimetric/fluorescent indicator	LOD 5.47 µM TMA; ~5 s optical response	Single-app quantification; freshness grading	[48]
E-nose for volatile amines (meat/fish)	Hybrid nanofibrous MOx mats; array + PCA	Distinguishes amines and spoilage stages (prototype)	Fiber mats integrated into e-nose; room-temp operation	[19]

Note: EU TVB-N limits (species-dependent) are 25/30/35 mg N per 100 g flesh; the reference method is applicable in the 5–100 mg/100 g range [49,50].

**Table 5:** Representative nano-enabled adulterant/contaminant sensors (quantitative examples).

Target (Matrix)	Nanomaterial and Transduction	Performance (LOD/Range/Time)	Notes	References
Melamine (milk)	AgNP colorimetry (aggregation)	LOD 0.04 mg L^−1^ (40 µg L^−1^); minutes	Unmodified AgNPs; visual/UV-Vis readout	[15]
Melamine (raw milk)	AuNP colorimetry (aggregation)	Visual LOD ≈ 0.07 mg L^−1^ (70 µg L^−1^); ~20 min	Naked-eye color change red→blue	[55]
Melamine (milk, smartphone)	Au@carbon quantum dots (Au@CQDs) smartphone readout	LOD 3.6 nM (~0.00045 mg L^−1^); LOQ 12 nM	Microarray + smartphone RGB; high sensitivity	[52]
Sudan I dye (sauces, powders)	Fe_3_O_4_ NPs @ GCE; DPV	LOD 0.001 µM (~0.248 µg L^−1^); linear 0.01–20 µM	Electrocatalytic oxidation; validated recoveries 96–104%	[16]
Aflatoxin B1 (cereals)	Au–Fe_3_O_4_ core–shell nano-immunosensor (EQCM-CV)	LOD 0.07 ng mL^−1^; 0.05–5 ng mL^−1^; reusable ~15×	Magnetically regenerable label; high sensitivity	[56]
Sucrose adulteration (juices)	PANI–Invertase–AuNP electrode (amperometric)	LOD 9 µM; good linearity	Enzyme entrapment within conductive polymer	[16]
Sucrose (juices; screen-printed)	PtNP–MWCNT enzyme nanocomposite (amperometric)	LOD 1 nM; wide linear range	Portable SPCE; validated vs. DNSA	[54]

Note: Codex maximum levels for melamine are 1 mg kg^−1^ (powdered infant formula) and 2.5 mg kg^−1^ (other foods/feeds); 0.15 mg kg^−1^ applies to liquid infant formula (2012 adoption). The LODs reported here are below these limits [54,57,58].

**Table 6:** Summary of key advantages of nanomaterials in sensory systems.

Advantage	Example Nanomaterial	Application Example
High sensitivity	Gold nanoparticles	Colorimetric melamine detection
Rapid response	CNTs, graphene	Electrochemical ammonia sensing
Antimicrobial activity	Silver, ZnO nanoparticles	Spoilage prevention in dairy packaging
Nutrient encapsulation	Nanoliposomes, nanoemulsions	Controlled release of omega-3 in beverages
Flexible integration	Graphene, nanocellulose	Smart labels for freshness indication

**Table 7:** Key future applications of nanotechnology in food monitoring.

Innovation Area	Future Potential Example	Supporting Nanomaterials
Multifunctional Smart Packaging	Real-time detection and antimicrobial response	ZnO, graphene oxide, nanocellulose
IoT-Connected Sensors	Cloud-based freshness monitoring	CNTs, wireless nanochips
Personalized Food Analysis	Smartphone-integrated allergen/nutrient detection	Gold nanoparticles, graphene
Edible Sensors	Sensors digestible with food for metabolic feedback	Biopolymer-based quantum dots

## Data Availability

No new data are created.

## Abbreviations

2D	two-dimensional (materials)
AgNP(s)	silver nanoparticle(s)
AuNP(s)	gold nanoparticle(s)
AI	artificial intelligence
CFU (mL^−1^)	colony-forming unit(s) (per milliliter)
CNT(s)	carbon nanotube(s)
CQD(s)	carbon quantum dot(s)
CV	cyclic voltammetry
DNSA (method)	3,5-dinitrosalicylic acid assay
DPV	differential pulse voltammetry
E-nose	electronic nose (sensor array + pattern recognition)
EFSA	European Food Safety Authority
EIS	electrochemical impedance spectroscopy
EQCM	electrochemical quartz crystal microbalance
FET	field-effect transistor
GCE	glassy carbon electrode
GRAS	Generally Recognized As Safe
IMS	immunomagnetic separation
IoT	Internet of Things
LOD	limit of detection
LOQ	limit of quantification
LSPR	localized surface plasmon resonance
MIP(s)	molecularly imprinted polymer(s)
MOx	metal oxide(s) (eg, ZnO, TiO_2_)
MWCNT(s)	multi-walled carbon nanotube(s)
NFC	near-field communication
NP(s)	nanoparticle(s)
PANI	polyaniline
PCA	principal component analysis
PEC	photoelectrochemical
PDA	polydiacetylene
PtNP(s)	platinum nanoparticle(s)
QD(s)	quantum dot(s)
RGB	red–green–blue (digital channels)
RFID	radio-frequency identification
SERS	surface-enhanced Raman scattering
SPCE	screen-printed carbon electrode
TMA	trimethylamine
TOC (figure/diagram)	table-of-contents figure/diagram
TTI	time–temperature indicator
TVB-N	total volatile basic nitrogen
UV–Vis	ultraviolet–visible (spectroscopy)
VOC(s)	volatile organic compound(s)
